# Supplementary material for: Inferred global dense residue transition graphs from primary structure sequences enable protein interaction prediction via directed graph convolutional neural networks
Source: Front Bioinform. 2025 Oct 22;5:1651623. doi: 10.3389/fbinf.2025.1651623 (PMC12585958; doi:10.3389/fbinf.2025.1651623)
Supplement: Supplementary file 1 [file DataSheet1.pdf]

Supplementary Material for ProtGram-DirectGCN

1 APPENDIX A: ALGORITHMS

This section provides a high-level summary of the four algorithms that constitute the $ProtGram-DirectGCN$ pipeline. The methodology is designed to create a hierarchical representation of proteins, starting from basic n-grams and culminating in comprehensive protein-level embeddings.

Algorithm 1: $ProtGram$: Hierarchical N-gram Graph Construction

This foundational algorithm builds a hierarchy of directed, weighted graphs $\{G_1, \ldots, G_{N_{max}}\}$ from a corpus of protein sequences $\mathcal{S}$. For each n-gram size n, the algorithm performs a single pass over the data to extract unique n-grams (nodes V_n) and their sequential co-occurrences (directed edges E_n). The weight of an edge from n-gram u to v quantifies the frequency of their transition. The output is a series of graphs where each G_n captures the relational structure of n-grams at a specific length scale.

Algorithm 2: $DirectGCN$: Layer Forward Pass

This algorithm specifies the forward pass for a single layer in the $DirectGCN$ model. It updates node features H through a sophisticated multi-path architecture. The process involves:

1. A **shared linear transformation** applied to the input features.
2. **Multi-path message passing**, where information is propagated and transformed independently across different edge types (e.g., based on in-degree, out-degree, or as an undirected graph).
3. A **hierarchical gating mechanism** that uses learnable weights to compute an adaptive, weighted sum of the outputs from all paths. This allows the model to dynamically prioritize different types of neighborhood information.

Algorithm 3: $DirectGCN$: Model Forward Pass

This algorithm outlines the complete forward pass for the full $DirectGCN$ model, which stacks multiple layers defined in Algorithm 2. The architecture integrates modern deep learning components, including **residual connections**, **Layer Normalization**, and **Dropout**, to facilitate stable training of a deep network. The model is adaptable, employing a deeper configuration for the full ProtGram pipeline and a simpler one for benchmark tasks. The final output provides both node-level embeddings (H_{final}) and classification logits ($\hat{Y}$) from a terminal MLP decoder.

Algorithm 4: $ProtGram - DirectGCN$: Hierarchical Training

This master algorithm orchestrates the end-to-end training and embedding generation process. It consists of two primary phases:

1. **Hierarchical GNN Training:** The algorithm iteratively trains a $DirectGCN$ model on each graph G_n from the hierarchy. For each level $n > 1$, the initial node features are derived by pooling the learned embeddings from the previous level $(n-1)$, establishing a curriculum where representations become progressively more complex. Each level is trained using a self-supervised task.
2. **Protein-Level Embedding Aggregation:** After the hierarchical training is complete, this phase generates the final embeddings for each full protein. It retrieves the learned representations for all

n-grams within a protein from the highest level (N_{max}) and aggregates them using an **attention pooling** mechanism. This produces a single, fixed-size vector representation for each protein in the corpus.

Algorithm 1 $ProtGram$: Hierarchical N-gram Graph Construction

Require: Corpus of protein sequences $\mathcal{S}$, Maximum n-gram size N_{max}
Ensure: A set of directed, weighted n-gram graphs $\{G_1, \ldots, G_{N_{max}}\}$

for $n = 1 \ldots N_{max}$ **do**
    Phase 1: Single-Pass N-gram and Edge Extraction
    Initialize unique n-gram set $V_n \leftarrow \emptyset$
    Initialize edge weights $W_n \leftarrow$ empty map
    for each protein sequence R in $\mathcal{S}$ **do**
        if length$(R) < n + 1$ **then** ▷ Skip sequences too short to form an edge
            continue
        end if
        for $i = 0 \ldots$ length$(R) - n - 1$ **do**
            $u \leftarrow R[i : i + n]$ ▷ Source n-gram
            $v \leftarrow R[i + 1 : i + 1 + n]$ ▷ Target n-gram
            Add u and v to V_n
            Increment weight for edge (u, v) in W_n
        end for
    end for

    Phase 2: Graph Assembly
    Create integer mapping $ID_n : V_n \rightarrow \{0, \ldots, |V_n| - 1\}$
    Create weighted edge list E_n from W_n using the mapping ID_n
    $G_n \leftarrow$ InstantiateGraph(V_n, E_n)
    Store G_n
end for
return $\{G_1, \ldots, G_{N_{max}}\}$

Algorithm 2 $DirectGCN$: Layer Forward Pass

Require: Node features H, Graph data object $\mathcal{D}$
Ensure: Updated node features H'

// Phase 1: Shared Feature Transformation
$H_{shared} \leftarrow \text{LinearShared}(H)$

// Phase 2: Multi-Path Message Passing
Initialize empty list H_{paths}
for each path p in $\{$in-degree, out-degree, undirected, etc.$\}$ **do**
  $H_{prop_p} \leftarrow \text{Propagate}(\mathcal{D}.E_p, \text{Linear}_p(H)) + \text{bias}_p$
  $H_{comb_p} \leftarrow \text{Project}_p(H_{prop_p} \oplus (H_{shared} + \text{bias}_{shared_p}))$ ▷ $\oplus$ is concatenation
  Add H_{comb_p} to H_{paths}
end for

// Phase 3: Hierarchical Gating and Aggregation
$W_{gates} \leftarrow \sigma(\text{LearnableGatingCoefficients } C)$ ▷ σ is the sigmoid function
$H' \leftarrow \sum_p W_{gates_p} \odot H_{paths_p}$ ▷ $\odot$ is element-wise product
$H' \leftarrow H' + \text{LearnableNodeConstant}$
return H'

Algorithm 3 $DirectGCN$: Model Forward Pass

Require: Initial node features X, Graph data object $\mathcal{D}$
Ensure: Logits for classification $\hat{Y}$, Final node embeddings H_{final}

$H \leftarrow X$
if model is in ProtGram context (deep architecture) **then**
  for $i = 0 \ldots \text{NumLayers} - 1$ **do**
    $H_{conv} \leftarrow \text{DirectGCNLayer}_i(H, \mathcal{D})$
    $H_{res} \leftarrow \text{ResidualProjection}_i(H)$
    $H \leftarrow \text{LeakyReLU}(\text{LayerNorm}_i(H_{conv} + H_{res}))$
    $H \leftarrow \text{Dropout}(H)$
  end for
else (model is in Benchmark/Singleton context)
  $H \leftarrow \text{DirectGCNLayer}_0(H, \mathcal{D})$
  $H \leftarrow \text{ReLU}(\text{LayerNorm}_0(H))$
  $H \leftarrow \text{Dropout}(H)$
end if
$H_{final} \leftarrow H$

// Final Classification Head
$\hat{Y} \leftarrow \text{DecoderMLP}(H_{final})$
return $\hat{Y}, H_{final}$

Algorithm 4 $ProtGram - DirectGCN$: Hierarchical Training

Require: Set of n-gram graphs $\{G_1, \ldots, G_{N_{max}}\}$, Protein sequences $\mathcal{S}$, GNN model type $\mathcal{M}$
Ensure: Final protein-level embeddings $E_{protein}$

```
Initialize map of embeddings per level E_levels ← ∅

// Phase 1: Hierarchical GNN Training
for n = 1 ... N_max do
    Load graph G_n
    if n = 1 then
        X_n ← RandomFeatures(num_nodes(G_n), dim_init)
    else
        X_n ← PoolEmbeddings(E_levels[n − 1], G_n, G_{n−1})      ▷ Pool from previous level
    end if
    Y_n, num_classes ← GenerateTaskLabels(G_n, task_type_n)      ▷ Self-supervised task
    M_n ← InstantiateModel(M, dim(X_n), num_classes)
    Train M_n on (G_n, X_n, Y_n) to minimize loss L
    E_levels[n] ← ExtractEmbeddings(M_n, G_n, X_n)               ▷ Get final node embeddings
end for

// Phase 2: Protein-Level Embedding Aggregation
Initialize protein embeddings E_protein ← empty map
for each protein sequence R in S do
    N_R ← GetAllNgrams(R, N_max)
    E_R ← LookupEmbeddings(N_R, E_levels[N_max])                 ▷ Get embeddings from final level
    E_protein[R_id] ← AttentionPool(E_R)
end for
return E_protein
```

2 A REVIEW OF CONVOLUTIONAL METHODS FOR DIRECTED GRAPHS

Extending Spectral Graph Convolutional Networks (GCNs) to directed graphs is a significant challenge, as the standard spectral GCN formulation relies on the symmetric Laplacian of an undirected graph. Research to address this has broadly followed three themes: adapting spectral theory, redesigning spatial convolutions, and leveraging higher-order structures.

Spectral approaches aim to redefine the graph Laplacian to handle directedness. An early attempt by Ma et al. (2019) constructed a symmetric Laplacian using the graph's transition matrix and its stationary distribution (the Perron vector), but this is computationally expensive. A more recent innovation is MagNet Zhang et al. (2021), which employs a complex Hermitian matrix called the magnetic Laplacian, $L_N^{(q)} := I - (D_s^{-1/2} A_s D_s^{-1/2}) \odot \exp(i\Theta^{(q)})$, where directionality is elegantly encoded in the phase of complex entries, guaranteeing real eigenvalues suitable for spectral filtering. Another important model is DirGNN Rossi et al. (2023) focuses on "heterophilic" graphs, where connected nodes likely have different labels or types, it contrasts with "homophilic" graphs, where neighboring nodes tend to share similar labels. The authors argue that most Graph Neural Networks (GNNs) overlook edge direction by converting directed

graphs into undirected forms. This means the direction of relationships is ignored, which can be unideal for heterophilic graphs. Dir-GNN addresses this by performing separate aggregations of information for incoming and outgoing edges, considering their direction. This approach improves effective homophily, or how well related information is grouped together, in datasets with many differing node labels. The research shows notable accuracy gains on benchmarks designed for heterophilic graphs, achieving top results by using edge direction while still performing well on homophilic graphs.

A second, more intuitive theme involves spatial approaches that redefine the neighborhood aggregation process. The most common strategy, seen in models like Tong et al. (2020)'s, is to explicitly separate the aggregation of features from in-neighbors and out-neighbors. A generalized form of this operation is $H' = \sigma(\tilde{A}_{in}HW_{in} + \tilde{A}_{out}HW_{out})$, where $\tilde{A}_{in}$ and $\tilde{A}_{out}$ are normalized adjacency matrices for incoming and outgoing edges. This method is straightforward to implement and scale but has a localized receptive field.

Finally, some research has moved beyond simple edges to consider higher-order connectivity patterns. MotifNet Monti et al. (2018), uses small, recurring directed subgraphs called motifs to define multiple anisotropic views of the graph, allowing the model to capture more complex structural information. Our $ProtGramDirectGCN$ builds upon the principles of spatial methods by explicitly handling incoming, outgoing, and undirected information channels. However, it is inherently spectral introducing a unique hybrid $DirectGCN$ layer architecture with a gating mechanism, allowing the model to learn the relative importance of each information channel and a shared feature transformation for a more expressive aggregation scheme.

3 DATA PREPARATION

4 DATA ACQUISITION AND PREPROCESSING

The data processing methodology minimizes modifications to public datasets before analysis. All data cleaning, filtering, and tokenization tasks occur within the main processing pipeline.

4.1 Data Sources

The primary datasets for this study were acquired from the following official sources:

- **Protein Sequences:** The UniProt Swiss-Prot (UniProt-SPROT) and UniRef50 databases served as the primary sources for all protein sequences.
- **Positive Protein-Protein Interactions (PPIs):** A comprehensive set of positive interactions was obtained from the BioGrid database (Version 4.4.248, file BIOGRID-ALL-4.4.248.mitab.zip).
- **Negative Protein-Protein Interactions (PPIs):** A high-confidence set of negative (non-interacting) protein pairs was obtained from the curated datasets provided by Trabuco et al. (2012).
- **Identifier Mapping:** The official UniProt ID mapping database (idmapping.dat.gz) was utilized to standardize all protein identifiers to their corresponding canonical UniProtKB accession numbers.

4.2 Generation of PPI Ground Truth Sets

A standardized ground truth dataset of positive and negative interactions was created for model training and evaluation.

1. **Identifier Standardization** To ensure consistency across sources, a local identifier mapping database was built. The UniProt idmapping.dat file was parsed to create a relational table. This table maps GeneID identifiers to their UniProtKB accession numbers.
2. **Processing of Positive Interactions** The BioGrid dataset was processed to generate the positive interaction set:
   - The BIOGRID-ALL-4.4.248.mitab file was parsed to extract the first two columns, representing Interactor A and Interactor B, which are primarily identified by GeneIDs.
   - These GeneID pairs, which uniquely identify genes in NCBI, were added to a MySQL database.
   - A database join was performed between the BioGrid interaction table and the UniProt ID mapping table. This converted each GeneID in the pairs to its corresponding UniProtKB accession number.
   - The resulting table of UniProtKB ID pairs was exported as the positive interaction set.
   - We have also implemented an automated version of this process in the main pipeline, which relies on Parquet files.
3. **Processing of Negative Interactions** The negative interaction set was generated by aggregating data from Trabuco et al. (2012):
   - The source files, which are already indexed by UniProtKB identifiers, were processed to extract the first two columns representing the non-interacting protein pair.
   - Data from all relevant species-specific files were combined into a single table.
   - A string parsing function was used to keep only the core UniProtKB accession number. This number is a unique protein identifier in the UniProt database.
   - The final, aggregated table of non-interacting pairs was exported as the negative interaction set.

Both positive and negative interaction sets were saved in Parquet format for efficient downstream access.

4.3 Dynamic Sequence Preprocessing

Protein sequence data was processed immediately prior to model input, ensuring that each pipeline component receives data in the required standardized format.

Sequence Cleaning: When a model component requests a sequence, the raw FASTA file is read. A filter removes any character not representing one of the 20 standard amino acids (A, C, D, E, F, G, H, I, K, L, M, N, P, Q, R, S, T, V, W, Y). This standardizes the alphabet for all models. To fit GPU and memory limits, sequences are truncated to a maximum of 10,000 characters.

Tokenization: The cleaned and truncated sequence is converted into smaller units, called tokens, according to the specific requirements of the downstream model.

EXPERIMENTAL DETAILS

The experimental setup is defined by a series of fixed hyperparameters for the primary embedding generation models and a defined search space for optimizing the downstream Protein-Protein Interaction (PPI) classifier.

Model Hyperparameters

The following tables detail the fixed hyperparameters used for the main embedding generation pipelines and the GNN benchmarking suite.

Table S1. Hyperparameters for the $ProtGram - DirectGCN$ Pipeline.

Parameter	Value
Max N-gram Size (N_{max})	3
DirectGCN Hidden Layer Dims	[512, 256, 128, 64]
1-gram Initial Feature Dim	512
Epochs per N-gram Level	500
Learning Rate	0.005
Dropout Rate	0.5
Weight Decay	0.0001
Protein Pooling Strategy	attention
Hierarchical Pooling Strategy	attention

Table S2. Hyperparameters for the GNN Benchmarking Suite.

Parameter	Value
Epochs	100
Learning Rate	0.01
Weight Decay	0.0005
GNN Hidden Channels	64
GNN Number of Layers	2
GAT Heads	2
ChebNet K	3

Table S3. Hyperparameters for the final PPI Evaluation MLP.

Parameter	Value
Cross-Validation Folds	3
Epochs	10
Batch Size	2048
Learning Rate	0.001
Dense Layer 1 Units	128
Dropout Rate 1	0.4
Dense Layer 2 Units	64
Dropout Rate 2	0.4
L2 Regularization	1.0e-5
Edge Embedding Method	concatenate

Hyperparameter Optimization Search Space

For the hyperparameter optimization (HPO) pipeline, we used Optuna to search for the best-performing MLP architecture for the downstream PPI classification task. The search was conducted on the embeddings generated by the $ProtGram - DirectGCN$ model. Table S4 details the search space for each hyperparameter.

Table S4. Search space for the Hyperparameter Optimization of the PPI Evaluation MLP.

Hyperparameter	Type	Range / Choices
MLP Learning Rate	Log-Uniform	[1.0e-5, 1.0e-2]
Dense Layer 1 Units	Integer	[32, 512] (step of 32)
Dropout Rate 1	Uniform	[0.1, 0.6]
Dense Layer 2 Units	Integer	[16, 256] (step of 16)
Dropout Rate 2	Uniform	[0.1, 0.6]
L2 Regularization	Log-Uniform	[1.0e-6, 1.0e-3]
Batch Size	Categorical	{512, 1024, 2048, 4096}

COMPUTATIONAL CAPACITY

The project was developed and evaluated across two distinct computational environments to ensure both functional correctness and scalability.

1. Development and Testing Environment (Mobile Workstation)

Initial development, prototyping, and unit testing were conducted on a high-performance mobile workstation. This environment was sufficient for debugging, feature implementation, and running the integrated test suite on smaller, sampled datasets.

- **Processor:** 13th Gen Intel® Core™ i9-13900H (2.60 GHz base frequency)
- **Memory (RAM):** 32.0 GB
- **System Type:** 64-bit Operating System, x64-based processor

2. Scalability and Evaluation Environment (Server Node)

All large-scale data processing, model training, and final performance evaluations were executed on a dedicated server node designed for high-throughput computing and GPU-intensive tasks. This machine's specifications were critical for handling the full sequence datasets and running the comprehensive benchmarking suite.

- **Processor:** Dual Intel® Xeon® Gold 6426Y CPUs, providing a total of 32 cores and 64 threads (2.5 GHz base, up to 4.1 GHz Turbo).
- **Memory (RAM):** 128 GB of DDR5 4800MHz RDIMM ECC memory.
- **GPU:** A single NVIDIA RTX 6000 Ada Generation graphics card with 48 GB of GDDR6 memory.
- **Primary Storage:** A high-performance RAID array of four 2 TB NVMe SSDs (8 TB total raw capacity) hosted on a Dell Ultra-Speed Drive PCIe card, used for the operating system and active computations.

- **Secondary Storage:** A RAID 1 array of two 1.92 TB Enterprise SATA SSDs (1.92 TB usable capacity) managed by a PERC H755 hardware RAID controller, used for data backup and staging.
- **Networking:** Equipped with both standard dual-port 1GbE networking and a high-performance quad-port 25GbE SFP28 adapter for fast data access.

REFERENCES

[Dataset] Ma, Y., Hao, J., Yang, Y., et al. (2019). Spectral-based Graph Convolutional Network for Directed Graphs. doi:10.48550/arXiv.1907.08990

[Dataset] Monti, F., Otness, K., and Bronstein, M. M. (2018). MotifNet: A motif-based Graph Convolutional Network for directed graphs. doi:10.48550/arXiv.1802.01572

[Dataset] Rossi, E., Charpentier, B., Di Giovanni, F., et al. (2023). Edge Directionality Improves Learning on Heterophilic Graphs

Tong, Z., Liang, Y., Sun, C., et al. (2020). Digraph Inception Convolutional Networks. In *Advances in Neural Information Processing Systems* (Curran Associates, Inc.), vol. 33, 17907–17918

Trabuco, L. G., Betts, M. J., and Russell, R. B. (2012). Negative protein–protein interaction datasets derived from large-scale two-hybrid experiments. *Methods* 58, 343–348. doi:10.1016/j.ymeth.2012.07.028

[Dataset] Zhang, X., He, Y., Brugnone, N., et al. (2021). MagNet: A Neural Network for Directed Graphs
